# A Rare Case of Acute Ischemic Pancreatitis and Duodenitis in the Setting of Cardiogenic Shock

**DOI:** 10.1155/crgm/2791133

**Published:** 2025-05-29

**Authors:** Binyamin Ravina Abramowitz, Nealansh Embre Gupta, Syeda Maleehah Ali, Bani Chander Roland, Daniel Anthony DiLeo

**Affiliations:** ^1^Department of Medicine, SUNY Downstate Health Sciences University, Brooklyn, New York, USA; ^2^Department of Gastroenterology and Hepatology, SUNY Downstate Health Sciences University, Brooklyn, New York, USA; ^3^Department of Gastroenterology and Hepatology, VA New York Harbor Healthcare System, Brooklyn, New York, USA

**Keywords:** acute pancreatitis, cardiogenic shock, duodenitis, esophagogastroduodenoscopy, ischemic pancreatitis

## Abstract

Acute ischemic pancreatitis and duodenitis are uncommon diagnoses that can affect patients with shock. As respiratory, cardiac, and neurological manifestations of shock are prioritized, resultant gastrointestinal (GI) related pathologies can be overlooked, leading to underdiagnosis of these conditions. In this report, we highlight the importance of recognizing acute ischemic pancreatitis and duodenitis in our description of a unique patient presenting with GI related complaints with underlying cardiogenic shock.

## 1. Introduction

Acute pancreatitis is quite prevalent as it results in more than 250,000 annual hospital admissions in the U.S [1]. Although alcohol consumption and gallstone disease are the two most common etiologies, medications, hypercalcemia, hypertriglyceridemia, trauma, malignancy, and toxins can also be potential identifiable triggers. It is rare for ischemia to be the primary identifiable cause of acute pancreatitis [2]. We present a unique case of acute ischemic pancreatitis and duodenitis in a patient with cardiogenic shock.

## 2. Case Presentation

A 60-year-old male with a past medical history of congestive heart failure with recovered ejection fraction, coronary artery disease status post coronary artery bypass graft, atrial fibrillation, and chronic kidney disease presented following three days of melena and intermittent epigastric pain associated with nausea and vomiting. His home medications included rivaroxaban, aspirin, furosemide, metolazone, metoprolol succinate, and insulin. The patient's vital signs revealed a blood pressure of 97/63 mmHg and heart rate of 77 beats per minute. Physical exam findings included epigastric tenderness, cool extremities, and jugular venous distension. Laboratory data showed a hemoglobin of 4.3 g/dL (baseline of 8 g/dL), creatinine 3.1 mg/dL (baseline 1.7 mg/dL), lactic acid 7.69 mmol/L, calcium 9.2 mg/dL, albumin 3.8 g/dL, white blood cell count 7.8 K/μL, serum glucose 328 mg/dL, lactate dehydrogenase 633 U/L, aspartate aminotransferase 300 U/L, and lipase 88 U/L.

**Figure 1:**
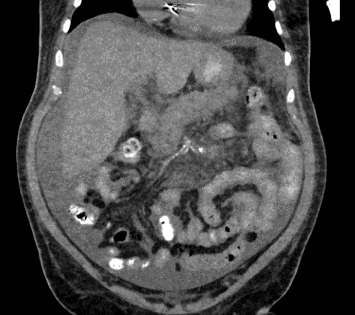
CTAP showing evidence of acute pancreatitis with peripancreatic inflammatory stranding.

**Figure 2:**
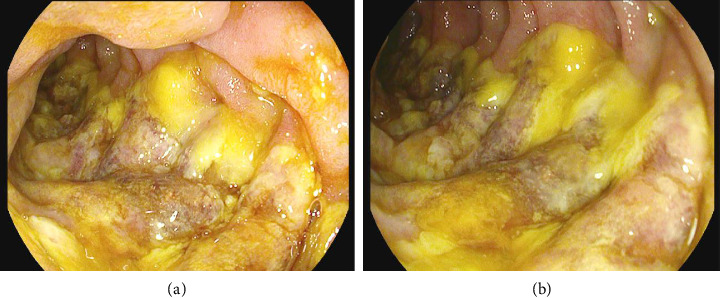
Endoscopic view of duodenal ischemia.

The patient was admitted to the medical intensive care unit (ICU), where he received 2 L of normal saline intravenously, four units of packed red blood cells, and andexanet alfa. As the patient was admitted for a life-threatening gastrointestinal (GI) bleed, the andexanet alfa was administered in an effort to reverse the blood-thinning effects of the rivaroxaban that the patient had taken as recently as the evening prior to his initial presentation. Computed tomography (CT) of the abdomen/pelvis demonstrated moderate peripancreatic inflammatory stranding without evidence of necrosis (Figure 1). Ultrasound of the abdomen did not demonstrate cholelithiasis. The patient denied any alcohol use. Serum alcohol level was negative and triglyceride level was normal. Echocardiogram demonstrated a new reduction in left ventricular ejection fraction to 35%. The patient underwent Esophagogastroduodenoscopy (EGD), which revealed a 6 cm hiatal hernia, esophagitis, and ulcerated mucosa in a linear distribution extending from the duodenal sweep through the second portion of the duodenum (Figure 2). It was determined that acute blood loss anemia was a result of ischemic duodenitis in the setting of cardiogenic shock. Given evidence of hypoperfusion and lack of alternate explanation, acute pancreatitis was also presumed to be the result of ischemia. Five days later, the patient required intubation and vasopressors for worsening cardiogenic shock. He experienced cardiac arrest and expired.

## 3. Discussion

Acute ischemic pancreatitis is not very common and is only documented within a handful of case reports [3]. Pancreatic ischemia may be the consequence of pancreatic hypoperfusion or even general dehydration and hypoxia [4]. In regard to pancreatic hypoperfusion, the extensive collateral blood supply to the pancreas typically renders it insusceptible to ischemia. The pancreatic head is perfused through the pancreaticoduodenal arteries from the superior mesenteric artery (SMA), and the body and tail are perfused through branches of the splenic artery from the celiac trunk [5]. Only a major, broader disruption of blood supply can interfere with this extensive network of collateral vessels to result in ischemia. In fact, the few reported cases of ischemic pancreatitis within the literature have been in settings such as aortic dissection [6–8], aortic thrombus [9], shock [10], and cardiac arrest [11].

Acute ischemic pancreatitis is challenging to recognize and diagnose as it often occurs simultaneously with dysfunction and failure of other organs. These patients are typically being treated in ICU where the management of their neurological, respiratory, and cardiac functional statuses are all prioritized, often leaving the state of their pancreas overlooked and sidelined [12]. Additionally, as ICU patients are often sedated or with altered mentation, clinical symptoms of acute pancreatitis may be masked, leading to underdiagnosis. Furthermore, to clinch the diagnosis of acute ischemic pancreatitis, it must be established that the setting of ischemia preceded the acute pancreatitis and not vice versa. Acute pancreatitis of all etiologies may result in secondary shock and ischemia, but in true acute ischemic pancreatitis, shock or ischemia must be the primary underlying pathology with acute pancreatitis as its sequelae. Therefore, all other potential etiologies of acute pancreatitis must be excluded before establishing a diagnosis of acute ischemic pancreatitis [13]. In our patient, all other etiologies of pancreatitis were excluded. Abdominal ultrasound did not reveal any cholelithiasis, CT imaging was not suspicious for pancreatic malignancy, lipid panel and calcium levels were within normal limits, the patient was not on any medications associated with pancreatitis, and he denied any history of alcohol intake.

Similarly, ischemic duodenitis is also uncommon. The blood supply of the duodenum is also highly collateral as it too is perfused by both branches of the SMA as well as branches of the splenic artery from the celiac trunk. Within the literature, cases of duodenal ischemia have been reported in the context of occlusive vascular disease, PEG tube placement complications, abdominal aortic aneurysms [14], transarterial chemoembolization, and shock [15] such as the cardiogenic shock experienced by our patient. Clinical symptoms of ischemic duodenitis include abdominal pain, melena, and hematemesis, and the diagnosis can be established with endoscopic evaluation of the duodenum [16]. As patients that may have ischemic duodenitis secondary to shock are typically too sick to undergo procedures, it can be challenging to obtain direct endoscopic visualization of an ischemic duodenum. As other causes of upper GI bleeding cannot be ruled out without endoscopic evaluation, ischemic duodenitis in the setting of shock is very difficult to diagnose.

As both ischemic pancreatitis and duodenitis are rare diagnoses, no standard set of recommendations or treatments for either condition exist. In most of the cases reported within the literature, patients were managed with bowel rest, intravenous fluid hydration, and pain control while the underlying cause of ischemia was addressed. Our patient was being treated with vasopressors in an attempt to manage the underlying cardiogenic shock, but unfortunately, the patient did not survive.

Simultaneous ischemic pancreatitis and duodenitis is exceptionally rare as both organs are perfused by various collateral vessels. Only a major disruption in general blood supply can result in these conditions. Our case highlights the importance of ischemic pancreatitis or duodenitis being on the differential when patients with hypotension and shock present with gastrointestinal symptoms such as abdominal pain, hematemesis, or melena.

## Data Availability

The data used to support this case report are included within the article.
